# Diffuse plasmacytoma of the pancreas: a rare entity

**DOI:** 10.1590/0100-3984.2016.0052

**Published:** 2017

**Authors:** Camila Soares Moreira de Sousa, Carla Lorena Vasques Mendes de Miranda, Marcelo Coelho Avelino, Breno Braga Bastos, Ilan Lopes Leite Mendes

**Affiliations:** 1 Med Imagem – Radiologia, Teresina, PI, Brazil.; 2 Hospital de Urgência de Teresina Prof. Zenon Rocha, Teresina, PI, Brazil.; 3 UDI 24 horas – Radiologia, Teresina, PI, Brazil.

**Figure 1:**
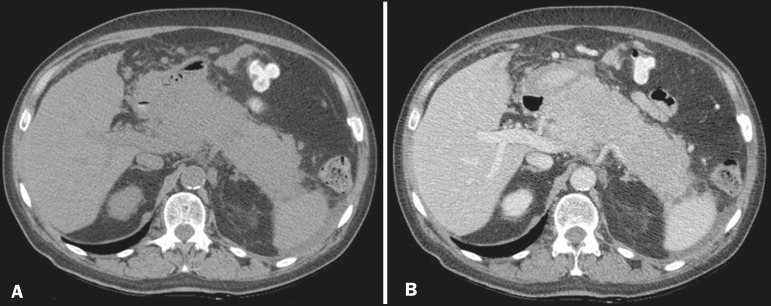
Axial CT scans of the abdomen, without contrast (A) and with contrast in the portal phase (B), showing diffuse, marked enlargement of the pancreatic parenchyma, with homogeneous uptake of the iodinated contrast medium.

**Figure 2:**
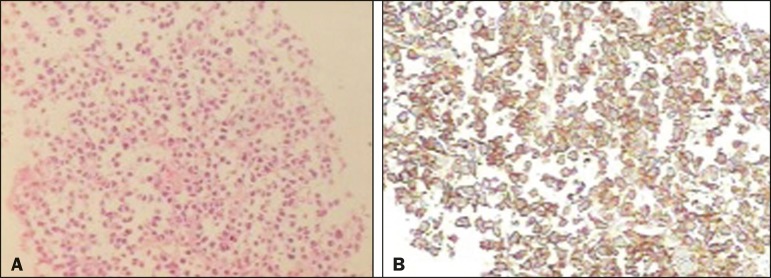
A: Histopathology showing malignant neoplasm composed of loosely cohesive atypical cells, with hyperchromatic, voluminous, eccentric nuclei, consistent with a diagnosis of plasma cell neoplasm. B: Immunohistochemistry showing CD138 expression, together with monoclonal immunoglobulin deposits of kappa light chain, confirming the diagnosis of pancreatic infiltration by plasmacytoma.

Dear Editor,

A 50-year-old male patient, diagnosed with multiple myeloma 10 months prior and undergoing chemotherapy, presented to the emergency department with abdominal pain. Laboratory tests revealed slightly elevated pancreatic enzymes. Subsequently, contrast-enhanced computed tomography (CT) of the abdomen showed diffuse, marked enlargement of the pancreatic parenchyma, with homogeneous uptake of the iodinated contrast medium in the portal phase (Figure 1). The initial working diagnosis was acute pancreatitis. However, the expected clinical, biochemical, and radiological improvement did not occur. We chose to perform CT-guided biopsy, and the histopathological analysis of the biopsy sample revealed a malignant neoplasm composed of loosely cohesive atypical cells, with hyperchromatic, voluminous, eccentric nuclei, consistent with a diagnosis of plasma cell neoplasm (Figure 2A). A complementary immunohistochemical study revealed expression of CD138, together with monoclonal immunoglobulin deposits of kappa light chain, confirming the diagnosis of pancreatic infiltration by plasmacytoma (Figure 2B).

Multiple myeloma is characterized by proliferation of malignant plasma cells originating from the bone marrow and accounts for 10% of all hematological malignancies. Extramedullary plasmacytoma accounts for 5% of all plasma cell tumors and primarily affects males, the mean age at presentation being approximately 55 years. They can be primary, occurring as solitary masses without bone marrow involvement, or secondary, occurring as part of a multiple myeloma, the latter being the more common presentation^(1-4)^. The most common site of extramedullary involvement is the upper respiratory tract (80%); however, other sites, such as the gastrointestinal tract, genitourinary tract, reticuloendothelial system, thyroid, lungs, skin, and testicles, can also be involved^(4)^.

There have been few reports of extramedullary plasmacytoma affecting the pancreas. Of the approximately 25 cases described, most have involved a focal mass and only one has involved diffuse infiltration of the pancreas^(2-6)^, ours therefore representing only the second such case reported. The most common site of presentation is the pancreatic head, in most cases resulting in abdominal pain and obstructive jaundice^(1-4)^. The radiological findings of pancreatic plasmacytoma are not highly specific. In the focal presentation, the solid mass is homogeneous or heterogeneous, multilobulated, with variable enhancement^(1)^; in the one previously reported case with a diffuse presentation, there was diffuse volumetric enlargement of the pancreas with lobulated contours and predominantly homogenous uptake in the portal phase^(6)^, similar to what was observed in the case reported here.

Although CT is the method of choice for the investigation of pancreatic plasmacytoma, it is not capable of excluding diseases such as adenocarcinoma, lymphoma, and metastasis, histopathology therefore being fundamental for the diagnosis^(3)^. In the case reported here, given the diffuse presentation, the main diagnostic hypotheses were pancreatitis and lymphoma. Lymphoma was excluded because of the clinical and laboratory findings, which indicated that pancreatitis was the most likely diagnosis. However, based on the history of multiple myeloma and the persistence of symptoms, the possibility of pancreatic infiltration by plasmacytoma was considered. Treatment for extramedullary plasmacytoma involves the combination of local radiation, chemotherapy, and, in selected cases, surgery^(4)^.

Plasmacytoma of the pancreas is a rare entity and continues to be the subject of many studies. In patients with multiple myeloma and focal or diffuse enlargement of the pancreas, the hypothesis of plasmacytoma should be considered, thus avoiding delayed diagnosis.
